# Pentraxin 3 (PTX3) as a Predictor of Severity of Sepsis in Patients Admitted to an Intensive Care Unit: A Cross-Sectional Study From North India

**DOI:** 10.7759/cureus.28282

**Published:** 2022-08-22

**Authors:** Kavya Ronanki, Mukesh Bairwa, Ravi Kant, Yogesh Bahurupi, Rajesh Kumar

**Affiliations:** 1 General Medicine, All India Institute of Medical Sciences, Rishikesh, IND; 2 Internal Medicine, All India Institute of Medical Sciences, Rishikesh, IND; 3 Community & Family Medicine, All India Institute of Medical Sciences, Rishikesh, IND; 4 College of Nursing, All India Institute of Medical Sciences, Rishikesh, IND

**Keywords:** serum procalcitonin, sofa score, serum pentraxin-3, serum lactate, severe sepsis

## Abstract

Background: Sepsis is a common clinical syndrome in critical patients in the medical intensive care unit. Many scoring systems and biomarkers are introduced to detect unfavorable outcomes in sepsis patients. This study aims to identify pentraxin 3 (PTX3) as a predictor of sepsis in patients who are critically ill and admitted to intensive care units.

Materials and methods: This prospective observational survey purposively included 100 patients with sepsis identified by the Surviving Sepsis Campaign guidelines in the medical intensive care unit at one of the apex care centers in North India. Socio-demographic and clinical profiles were collected using a structured and validated checklist. Simple and multi-linear regression analyses were used to determine PTX3 as a predictor of sepsis.

Results: A total of 100 patients were prospectively observed. Among them, 61% were males, and 39% were females, with a mean age of 50.78 (±13.53) years. From nine potential predictors, lactate (95% CI: 1.048-1.890, B: 1.469, p < 0.001), procalcitonin (95% CI: 0.136-0.270, B: 0.203, p < 0.001), and SOFA (Sequential Organ Failure Assessment) scores (95% CI: 0.112-0.450, B: 0.281, p = 0.001) significantly predict the changes in PTX3 level (R-square: 0.842, adjusted R-square: 0.826) in patients.

Conclusions: PTX3 was found to correlate with the severity of sepsis as SOFA score and other markers like lactate, procalcitonin, and APACHE-II (Acute Physiology and Chronic Health Evaluation II) score.

## Introduction

Sepsis is one of the most common clinical syndromes caused by systemic infection and often leads to a lethal outcome in critically ill patients. The Global Sepsis Alliance recommendation defined sepsis as a condition of life-threatening organ dysfunction caused by the dysregulated host response to infection. It has been a significant cause of intensive care unit admission worldwide. Sepsis results from the complicated interactions between the host immune system and infecting viruses and bacteria [1]. The mortality associated with sepsis is significant around the globe and is more than breast and lung cancer altogether. Furthermore, a lack of specific treatment for sepsis results in higher incidence and many complications, including septic shock, multiple organ dysfunction syndrome, and death. Multiorgan dysfunction and septic shock are the most common cause of mortality in patients with sepsis [2].

Further, diagnosing sepsis remains a significant challenge for health professionals considering concurrent organ support, organ dysfunction, treatment before admission, and lack of a "gold standard" diagnostic test. Over the advancement in medical sciences, many scoring systems were introduced as a surrogate for organ dysfunction risk prediction for patients with proven or suspected infection, including SOFA (Sequential Organ Failure Assessment) and APACHE-II (Acute Physiology and Chronic Health Evaluation II), to determine the degree of organ dysfunction and severity of disease in chronically ill patients [3-5]. Likewise, many biomarkers have been proposed for risk prediction in severely ill patients. Procalcitonin (PCT) [6], serum lactate [7], and cytokines are studied in patients with sepsis and shock. The inflammatory process in the body leads cytokines to produce higher PCT from the liver and mononuclear cells and subsequently increases the level in the body [8].

Higher levels of plasma lactate have been considered an essential indicator of hemodynamic stability. Breakthrough work has been conducted on the role of serum lactate on the survival of critically ill patients, reflecting a higher level of lactate decreases survival. Earlier research on hemodynamically stable patients reported a higher lactate level in non-survivors of emergencies. A higher lactate level is common among chronically ill patients and is recommended as a reliable marker of illness severity and death [9,10].

Pentraxin 3 (PTX3) is an acute-phase protein that represents the subfamily of long pentraxin [11]. It has been found to have a strong association with the severity of infection and inflammation. The inflammatory process initiates the secretion of PTX3 in monocytes, endothelial cells, and dendritic or neutrophils [12].

Numerous studies done to date have noticed that PTX3 has an excellent diagnostic value in sepsis. PTX3 as a biomarker of sepsis, and its diagnostic utility in northern Indian tertiary care setup, would help us evaluate the future scope in patients with sepsis. A crunch of studies, none to the best of our knowledge, in the Indian subcontinent further emphasizes the need to assess the diagnostic utility of PTX3 in patients with sepsis. Therefore, we decided to conduct a survey to correlate serum PTX3 levels with the severity of sepsis.

## Materials and methods

Study design and setting

This is a cross-sectional study conducted over 18 months at All India Institute of Medical Sciences (AIIMS) Rishikesh, a tertiary healthcare center in Uttarakhand, India.

Study participants and sampling

**Figure 1:**
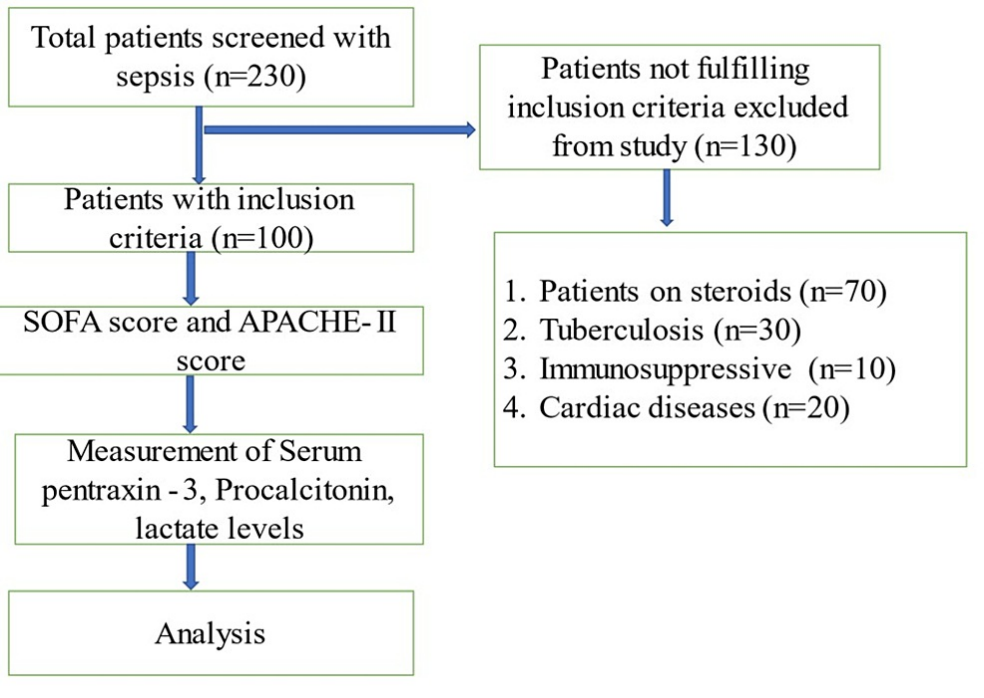
Patient enrollment in the study SOFA: Sequential Organ Failure Assessment; APACHE-II: Acute Physiology and Chronic Health Evaluation II.

The minimum sample size required for the study was estimated by using Fisher’s transformation formula, which came out to be 95. However, the authors decided to enroll 100 patients consecutively at the in-patient department of general medicine. The patients fulfilling the Surviving Sepsis Campaign guidelines and willing to write consent were included till the sample size was achieved. Patients who were on steroid therapy, diagnosed with immunodeficiency disorders, pregnant, and diagnosed with tuberculosis and acute coronary artery disease were excluded from the study (Figure 1).

Data collection tools and techniques

Socio-demographic details and baseline investigations were recorded using a structured and validated checklist. SOFA score and APACHE-II scores were calculated. PCT was measured by chemiluminescence on the Advia Centaur instrument (Siemens Healthineers, Erlangen, Germany), and lactate was measured by arterial blood sample using a blood gas analyzer (ABL800, Radiometer, Copenhagen, Denmark). Blood samples were collected into plain and ethylenediaminetetraacetic acid (EDTA) vials. All the blood samples were subjected to centrifugation at 2500×g for 10 minutes at 4°C within 30 minutes of blood sampling. Plasma and serum got separated and aliquoted. The aliquoted samples were collected and stored until analysis at -80°C. Pentraxin was analyzed by Sandwich ELISA (ImmunoTag, St. Louis, MO) following the manufacturer’s instruction after obtaining a satisfactory standard curve.

Ethical consideration

The Institutional Ethics Committee of All India Institute of Medical Sciences Rishikesh (AIIM/IEC) approved the project (AIIMS/IEC/20/575). Written informed consent was obtained from each participant before enrolling in the study. Participants were ensured to protect privacy and confidentiality at each stage of research.

Data analysis

Data were transferred to a Microsoft Excel sheet (Microsoft Corporation, Redmond, WA) and analyzed by using Statistical Package for the Social Sciences (SPSS) version 26.0 (IBM Corp., Armonk, NY). Frequency, percentage, means, and standard deviation (SD) were used to describe the patients’ characteristics. Categorical variables were represented as proportions. Continuous or discrete variables were reported using means and SD. Spearman correlation coefficient was used to find the correlation between PTX3 and other continuous variables considering the non-normal distribution of the variables. Mann-Whitney U test was used to compare the distribution of mean PTX3 levels with genders. Simple and multivariate regression analyses were done to find out whether PTX3 is a predictor of sepsis level. All test statistics were measured at p < 0.05 level (two-tailed).

## Results

A total of 100 participants were included in the study; among them, 61.0% were males and 39% were females. The mean age of the patients was 50.78 (SD = 13.53) years. Of the participants, 63% had one or more comorbidities, 21% had only diabetes mellitus, 21% had only hypertension, and 21% had both hypertension and diabetes mellitus (Appendix A). The mean SOFA score was 8.94 (SD = 2.86), the mean APACHE-II score was 14.48 (SD = 5.05), and the mean PXT3 value was 5.24 (SD = 3.38) ng/ml. Further, the PXT3 level did not show any significant difference with comorbidity status (p = 0.134) (Appendix B). Similarly, results show no significant difference in PXT3 levels among males and females (U = 982, p = 0.142). Serum lactate (r = 0.661, p < 0.001) and PCT (r = 0.663, p < 0.001) reported significant association with SOFA scores (Table 1).

**Table 1:** Laboratory parameters of the participants (n = 100) * Significant at 0.05 level; ** significant at 0.01 level. APACHE-II: Acute Physiology and Chronic Health Evaluation II; SD: standard deviation, p-value < 0.05; SOFA: Sequence Organ Failure Assessment Score; HR: heart rate; RR: respiratory rate: INR: international normalized ratio; TLC: total leucocytes count; HCO3: bicarbonate; FiO2: fraction of inspired oxygen; PaO2: partial pressure of oxygen; ICU: intensive care unit; PCO2: partial pressure of carbon dioxide; PCT: procalcitonin; SGOT: serum glutamic oxaloacetic transaminase; SGPT: serum glutamic pyruvic transaminase.

Parameter	Mean	SD	Test statistics	P-value
Pentraxin 3 (ng/ml)	5.24	3.38	1	1
pH (units)	7.4	0.12	-0.221^*^	0.027*
FiO2 (%)	45.31	18.71	0.267^**^	0.007*
PaO2 (mm Hg)	82.42	19.82	-0.424^**^	<0.001
PCO2 (mm Hg)	40.01	12.62	0.263^**^	0.008*
HCO3 (mmol/l)	20.92	5.91	0.032	0.754
Lactate (mmol/l)	2.63	1.01	0.823^**^	<0.001
PCT (ng/ml)	9.72	6.41	0.856^**^	<0.001
HR (min)	117.7	12.92	-0.026	0.797
RR (min)	25.54	4.13	0.093	0.356
TLC (cells/mm3)	17802.12	11686.8	0.114	0.258
Platelets (cells/mm3)	121078.42	120947.5	-0.151	0.133
Urea (mg/dl)	67.59	50.99	0.215^*^	0.032
Creatinine (mg/dl)	2.02	2.27	0.193	0.054
Total bilirubin (mg/dl)	1.62	2.88	0.192	0.056
SGOT (U/L)	86.76	100.74	-0.005	0.961
SGPT (U/L)	75.64	109.37	0.094	0.354
INR	1.67	1.12	0.113	0.261
SOFA score	8.94	2.86	0.722^**^	<0.001
APACHE-II	14.48	5.05	0.393^**^	<0.001

Considering the non-normal distribution of PXT3 among patients, Spearman’s rho test was applied to find an association with the SOFA scores. The findings show a significant positive correlation of PXT3 with the fraction of inspired oxygen (FiO2), partial pressure of carbon dioxide (PCO2), lactate, PCT, and blood urea. Further, the PXT3 level reported a significant positive relationship with the SOFA score (r = 0.722) and APACHE-II scores (r = 0.393) (Table 2).

**Table 2:** Non-parametric correlations of different variables with pentraxin 3 * Significant at 0.05 level; ** significant at 0.01 level. APACHE-II: Acute Physiology and Chronic Health Evaluation II; SBP: systolic blood pressure; DBP: diastolic blood pressure; HCO3: bicarbonate; BP: blood pressure; SGOT: serum glutamic oxaloacetic transaminase; SGPT: serum glutamic pyruvic transaminase; SD: standard deviation, p-value < 0.05; SOFA: Sequence Organ Failure Assessment Score; HR: heart rate; RR: respiratory rate; INR: international normalized ratio; TLC: total leucocytes count; PCO2: partial pressure of carbon dioxide; PCT: procalcitonin; FiO2: fraction of inspired oxygen; PaO2: partial pressure of oxygen; ICU: intensive care unit.

Variables	Test statistics	P-value
Age (years)	0.142	0.161
SBP (mm Hg)	-0.138	0.172
DBP (mm Hg)	-0.043	0.667
Mean BP (mm Hg)	-0.055	0.585
pH	-0.221^*^	0.027*
FiO2 (%)	0.267^**^	0.007*
PaO2 (mm Hg)	-0.424^**^	<0.001
PCO2 (mm Hg)	0.263^**^	0.008*
HCO3 (mmol/l)	0.032	0.754
Lactate (mmol/l)	0.823^**^	<0.001
PCT (ng/ml)	0.856^**^	<0.001
HR (min)	-0.026	0.797
RR (min)	0.093	0.356
TLC (cells/mm3)	0.114	0.258
Platelets (cells/mm3)	-0.151	0.133
Urea (mg/dl)	0.215^*^	0.032
Creatinine (mg/dl)	0.193	0.054
Total bilirubin (mg/dl)	0.192	0.056
SGOT (U/L)	-0.005	0.961
SGPT (U/L)	0.094	0.354
INR	0.113	0.261
SOFA. score	0.722^**^	<0.001
APACHE-II	0.393^**^	<0.001

**Figure 2:**
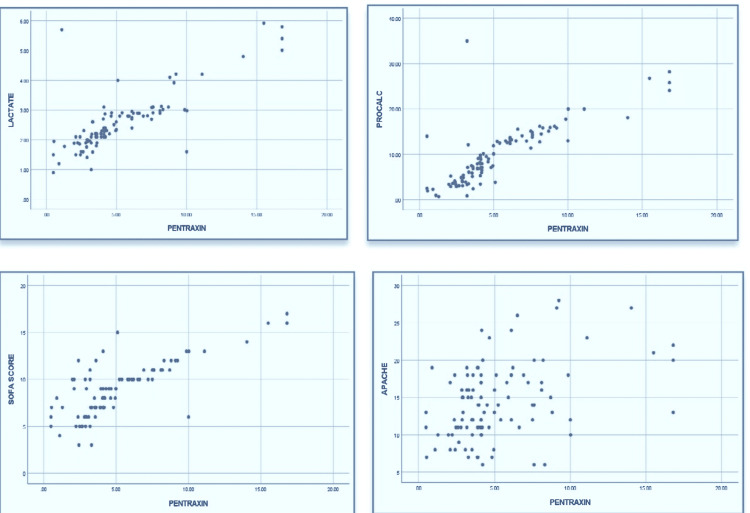
Scatter plots for correlation between pentraxin 3 and lactate, procalcitonin, SOFA score, and APACHE-II score SOFA: Sequence Organ Failure Assessment Score; APACHE-II: Acute Physiology and Chronic Health Evaluation II.

Simple linear regression analysis depicted that out of nine potential variables, seven variables, including FiO2 (p < 001), partial pressure of oxygen (PaO2) (p = 0.004), PCO2 (p = 0.034), PCT (p < 0.001, R-square, 0.657), lactate (R-square, 0.635), SOFA (p < 0.001, R-square 0.610), and APACHE-II (p < 0.001), highly predicted the change in PXT3 (Table 3 and Figure 2).

**Table 3:** Predictors of pentraxin 3: simple linear regression coefficients * p < 0.05; Beta: standardized coefficients; B: unstandardized coefficients; CI: confidence interval; PCT: procalcitonin; FiO2: fraction of inspired oxygen; PaO2: partial pressure of oxygen; PCO2: partial pressure of carbon dioxide; SOFA: Sequence Organ Failure Assessment Score; APACHE-II: Acute Physiology and Chronic Health Evaluation II.

Predictors/independent variables	B	Beta	P-value	95% CI	Regression results	
					R	R-square
pH	-9.84	-0.18	0.059	-20.06 to 0.38	0.189	0.036
FiO2	0.063	0.346	<0.001>	0.02-0.09	0.346	0.119
PaO2	-0.048	-0.283	0.004*	-0.08 to -0.01	0.283	0.080
PCO2	0.057	0.212	0.034*	0.004-0.110	0.212	0.045
Lactate	2.800	0.797	0.000*	2.374-3.226	0.797	0.635
PCT	0.429	0.811	0.000*	0.367-0.491	0.811	0.657
Urea	0.010	0.151	0.133	-0.003 to 0.02	0.151	0.023
SOFA	0.923	0.781	<0.001	0.775-1.071	0.781	0.610
APACHE-II	0.277	0.413	<0.001	0.154-0.399	0.413	0.171

Multivariate linear regression analysis was used to combine the cumulative effect of significant variables of simple linear regression. Findings reported that lactate (95% CI: 1.048-1.890, B: 1.469, p < 0.001), PCT (95% CI: 0.136-0.270, B: 0.203, p < 0.001), and SOFA score (95% CI: 0.112-0.450, B: 0.281, p = 0.001) significantly predict the change in PXT3 level (R-square: 0.842; adjusted R-square: 0.826) (Table 4).

**Table 4:** Predictors of pentraxin 3: multivariate linear regression coefficients * p < 0.05; Beta: standardized coefficients; B: unstandardized coefficients; CI: confidence interval; FiO2: fraction of inspired oxygen; PaO2: partial pressure of oxygen; PCO2: partial pressure of carbon dioxide; SOFA: Sequence Organ Failure Assessment Score; APACHE-II: Acute Physiology and Chronic Health Evaluation II.

Predictors/independent variables	B	Std. error	Beta	t-value	P-value	95% CI	
Constant	-44.891	19.305		-2.325	0.022	-83.243	-6.539
FiO2	0.012	0.009	0.064	1.277	0.205	-0.006	0.030
PaO2	-0.002	0.008	-0.010	-0.231	0.818	-0.017	0.013
PCO2	0.0013	0.013	0.049	1.023	0.309	-0.012	0.038
Lactate	1.469	0.212	0.418	6.932	<0.001	1.048	1.890
pH	5.561	2.568	0.107	2.166	0.033*	0.459	10.66
Pro-Cal	0.203	0.034	0.384	5.989	<0.001	0.136	0.270
SOFA score	0.281	0.085	0.238	3.296	0.001*	0.112	0.450
APACHE-II	-0.009	0.035	-0.014	-0.265	0.791	-0.080	0.061
B-urea	0.001	0.003	0.019	0.396	0.693	-0.005	0.007

## Discussion

Sepsis is a major cause of mortality worldwide, especially in developing countries, including India, considering the lack of protocolized care and crunch of resources [13]. It remains the major cause of mortality among critically ill patients. It has been demonstrated in previous work that early identification and protocol-based treatment of severe sepsis can improve the survival of patients. In recent years, there are many novel biomarkers, including PTX3, C-reactive protein (CRP), PCT, and plasma PTX3, that are identified to early anticipate sepsis and plan treatment [14]. However, none of the single biomarkers is ideal and helpful in identifying critically ill patients with their respective drawbacks. This study analyzed the PTX3 level in critically ill patients with sepsis and its correlation with lactate and PCT and critical illness index, including SOFA score and APACHE-II score.

The mean age of participants (n = 100) was 50.78 (±13.524) years with a predominantly male population. The proportion of participants with either hypertension or diabetes only was equal. The mean arterial blood pressure of the patients was 67.01 (±10.518) mmHg. Most patients had lungs as the source of infection, followed by abdominal infection and urinary tract infection. These findings were in line with most of the trials, including those by Chatterjee et al., which were done in India where 53% of participants had lungs as the primary source [15]. Other sites included skin, bacteremia, etc., which were less commonly seen in our setting. Of 100 participants, only 68 had positive cultural results. Most of them had gram-negative bacteria in their culture, which included *Acinetobacter*, *Pseudomonas*, and *Klebsiella* species.

The relevant laboratory parameters were measured, and the SOFA score and APACHE-II score were calculated. We analyzed the relationship between PTX3 and various laboratory parameters. We observed that blood pH and PaO2 have a negative correlation with PTX3, whereas FiO2, PCO2, lactate, PCT, and blood urea had a positive correlation with PTX3.

The primary objective of the study was to analyze PTX3 levels and see the correlation with SOFA scores. The study findings reported that lactate and PCT show a significant correlation with PTX3 in the studied cohort. These findings are in accord with several previously published studies that reported a significant relationship of PTX3 with lactate and PCT [16,17].

The Third International Consensus Definitions for Sepsis and Septic Shock (Sepsis-3) recommended measuring lactate levels for septic shock [18]. It has been suggested that serum lactate levels can be used to screen for sepsis among adults with clinical suspicion of sepsis. Several studies were conducted to assess the use of lactate in this context [19,20]. We have also included lactate in our study, in which lactate was found to correlate with SOFA score, which is similar to the study done by Liu et al. in 2019 [16]. In our study, we have also found that PTX3 is positively correlated with lactate (p = 0.000). This is in line with a previous prospective study done by Hu et al. [14]. However, serum lactate alone is neither sensitive nor specific to rule in or rule out the diagnosis of sepsis on its own. The testing of lactate may not be available in resource-limited settings [21]. Hence, it was given as a weak recommendation to use lactate levels in serum as an adjunctive test to detect the probability of sepsis in patients with suspected but not confirmed sepsis.

PCT fulfills the need for high diagnostic accuracy in detecting sepsis, which is needed to be used as a biomarker, especially in comparison to conventional. PCT alone cannot identify specific pathogens of sepsis, but the level of PCT might be useful to estimate the probability of severe bacterial infection [22]. We measured the level of PCT of all the participants, the mean of which was 9.7 (SD: 6.4), and it was found to correlate with the SOFA score.

A prospective study done by Sudhir et al. depicted that there was a significant association between PCT and SOFA score [23]. But in a retrospective cohort study done by Yunus et al., there was a weak correlation between PCT and SOFA score [24]. In our study, PTX3 has a significant positive correlation with PCT with a p-value of 0.000 (r = 0.856), which is similar to the previous study [25].

APACHE-II score is one of the scoring systems used to determine the severity of disease and predict the mortality of sepsis patients. Hill et al., in a pilot study, found that PTX3 levels were increased in patients with sepsis and are related to APACHE-II scores when plotted according to the APACHE-II score quartile [26]. Our study found that PTX3 levels are correlating significantly with the APACHE-II score (p = 0.00).

We did multistep-wise forward linear regression analysis and interestingly we found that the best individual marker to predict PTX3 is PCT with an R-square of 0.657 and an adjusted R-square of 0.654, with p = 0.00. The predictability increased with PCT and lactate together with an R-square of 0.808 and an adjusted R-square of 0.804 significantly (p = 0.00). The predictability of PTX3 is further increased with PCT, lactate, and SOFA scores together significantly with an R-square of 0.830 and an adjusted R-square of 0.825.

The efficacy of PTX3 as a biomarker tool in sepsis has been demonstrated in the work of many studies. In the Albumin Italian Outcome Sepsis (ALBIOS) trial, which is a multi-centric trial done on 1818 patients, it was found that PTX3 levels are elevated in severe sepsis and correlate significantly with prevalent and incident organ failures [27]. Similarly, in a prospective study done by Uusitalo-Seppälä et al., it was found that measuring PTX3 level at admission highly predicts severe sepsis and case fatality [28]. Lee et al., in a meta-analysis, found that PTX3 significantly predicts the severity of the disease and mortality in sepsis [29].

Hamed et al. conducted the Mannheim Sepsis Study, which is a prospective, monocentric study done on 217 intensive care unit patients, included according to the latest Sepsis-3 definitions. This study demonstrated that PTX3 exhibits potential diagnostic value in comparison to CRP and interleukin-6, and PTX3 is correlating with SOFA score [12].

Albeit, PCT is a promising marker of infection and due to its early rise and short half-life, the studies are heterogeneous and lack consensus. Lactate is a marker detecting endogenous catecholamine release. Patients who are maintaining their blood pressure due to a vigorous catecholamine response may have deceptively reassuring vital signs and mask the catecholamine-dependent shock. Elevated lactate identifies these patients having occult shock who are more at risk of adverse outcomes so each of the three biomarkers plays a crucial role in helping the management of sepsis.

Akin to most of the studies, we found that PTX3 correlates with disease severity scores SOFA and APACHE-II. PTX3 is also correlating significantly with PCT and lactate, biomarkers found to be effective in sepsis for ages. We also found that PCT, lactate, and SOFA scores together predicted PTX3 significantly, and the predictability is better than individual components. Among PCT, lactate, and SOFA score, the best predictor of PTX3 is PCT. PTX3, in combination with established other markers, might improve the correlation with sepsis severity and needs to be studied.

Hence, the novel marker PTX3 with its advantages needs to be considered and to be studied in future studies, as sepsis is one of the most common causes of mortality in humankind, which should be emphasized.

This is a single-center study, and we have not included the mortality data; hence, an association between PTX3 and mortality cannot be established. In this survey, we measured PTX3 once, which may not be sufficient to conclude, and the authors recommend a longitudinal large-scale study to understand the exact role of PTX3 in sepsis development and other health consequences. A multi-centric randomized controlled trial might recheck the results of the present work. A study with a higher sample size is recommended to improve generalizability over other similar populations.

## Conclusions

Currently, the accuracy of various biomarkers in sepsis and septic shock and their correlation with severity has thrown open wide conflicting results with many studies showing different results. PTX3 was analyzed and studied according to the latest Sepsis-3 guidelines in our study and was found to correlate with the severity of sepsis as SOFA score and other markers like lactate and PCT along with APACHE-II score. Our study did not permit us to rush to the conclusion of whether PTX3 is a better marker compared to other biomarkers in sepsis in use now. It raised the question of whether PTX3 can be used as a tool in sepsis for early detection. To find a definitive answer, larger randomized control trials are needed.

The study correlated PTX3 levels with PCT and lactate, which are age-old markers established in sepsis, unlike other new markers. Second multi-stepwise forward linear regression analysis was done to see better predictors of PTX3, adjunctively demonstrating its correlation with sepsis. Henceforth, novel biomarkers such as PTX3 in combination with lactate, PCT, and SOFA score might be helpful to improve the risk stratification of patients with sepsis.

## Figures and Tables

**Figure 3:**
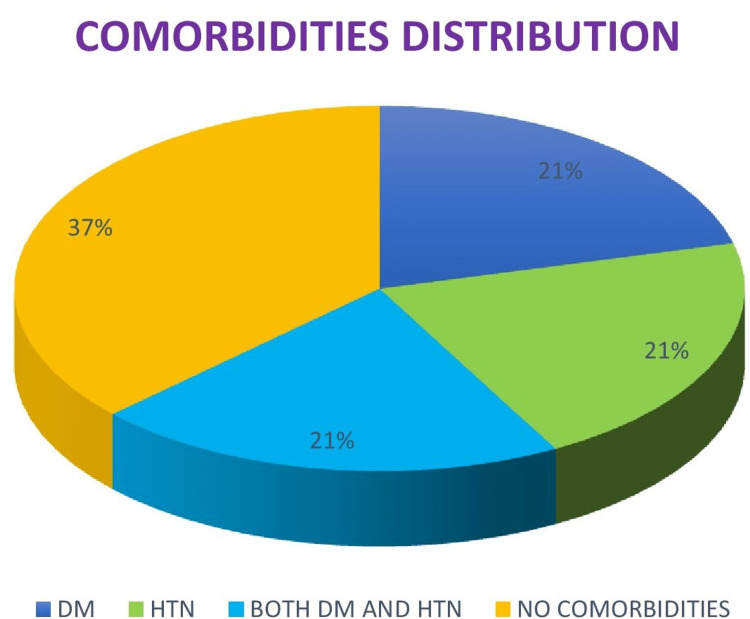
Comorbidities distribution DM: diabetes mellitus; HTN: hypertension.

## Appendices

Appendix A

Appendix B

**Table 5:** Mean level of pentraxin 3 in different comorbidities DM: diabetes mellitus; HTN: hypertension.

	Comorbidities	N	Mean rank	Chi-square	P-value
Pentraxin 3	DM	21	29.57	4.019	0.134
	HTN	21	38.48		
	Both	21	27.95		
	Total	63			
